# Analysis of social networks supporting the self-management of type 2 diabetes for people with mental illness

**DOI:** 10.1186/s12913-015-0897-x

**Published:** 2015-07-04

**Authors:** Mikaila M. Crotty, Julie Henderson, Paul R. Ward, Jeffrey Fuller, Anne Rogers, Debbie Kralik, Sue Gregory

**Affiliations:** School of Nursing & Midwifery, Flinders University, GPO Box 2100, Adelaide, 5001 Australia; Discipline of Public Health, Flinders University, GPO Box 2100, Adelaide, 5001 Australia; Faculty of Health Sciences, University of Southampton, Building 67, Highfield Campus, University Road, S017 1BJ Hampshire, UK; RDNS South Australia, Keswick, SA 5035 Australia

**Keywords:** Chronic illness self-management, Social networks, Mental health, Type 2 diabetes, Health services

## Abstract

**Background:**

People with mental illness have been identified as being more likely to experience type 2 diabetes and the complications arising from this, necessitating more complex chronic illness self-management. Social support has been identified as a significant factor in the successful adoption of lifestyle change for people with type 2 diabetes, however people with mental illness often have impoverished social networks leading to greater reliance upon professional care givers. This study maps the support provided by formal (paid and professional carers) and informal networks to people with mental illness and type 2 diabetes, comparing the experiences of people with a spouse with those without one.

**Methods:**

Interviews were conducted with 29 clients of a community nursing service with mental health problems who receive professional support to self-manage type 2 diabetes. Participants were asked to complete an egocentric social network map which involved mapping the people and services who support them to manage their health. Demographic data was collected as was data about co-morbidities and service use within the last 6 months. Network maps were supplemented by a series of open-ended questions about self-management practices, who supports these practices and what support they provide.

**Results:**

Participants identified small social networks with few friendship ties. These networks had diminished due to illness. For people with a spouse, this person provided significant support for chronic illness self-management performing a range of daily care and illness management tasks. People without a spouse were more reliant on professional and paid care givers for daily care and illness management. People without a spouse also demonstrated greater reliance upon weak social ties for emotional support and social connection and often developed friendships with formal caregivers.

**Conclusions:**

Spousal support reduces the need for professional services. In the absence of a spouse, participants were more reliant upon paid and professional carers and weaker social ties for chronic illness support and social connection leading to greater vulnerability of loss of support.

**Electronic supplementary material:**

The online version of this article (doi:10.1186/s12913-015-0897-x) contains supplementary material, which is available to authorized users.

## Background

This study explores the social networks of people with mental health problems who are also self-managing type 2 diabetes. Social networks have been defined as the “collection of interpersonal ties that people of all ages maintain” ([1], p.516). Social networks may have both a positive and negative impact on health. Members of social networks frequently demonstrate similar health risk behaviours. This has been attributed to contagion arising from the spread of information and behavioural norms within groups [2]; homophily (forming ties to similar others) [3]; and structural inequalities that can lead to poorer access to resources [4]. Conversely, social networks may promote health through providing access to social and cultural capital in the form of people, information and resources [5, 6]. Members of social networks may also promote referral to health services [7] and can be a source of social and physical support [8–10].

There is an emerging literature which demonstrates a link between mental illness and poorer physical health [11–13]. Iacovidis and Siamouli found that 50 % of people with mental illness have physical co-morbidities [12] with Fenton and Stover reporting an increased odds ratio for type 2 diabetes for people with depression compared with people without (OR = 2.2) [14]. Major depression is also linked to higher mortality rates from type 2 diabetes (OR = 2.3) [14] and is associated with diabetes related complications such as ulcers and hyperglycaemia [15]. People with mental and physical health comorbidities often have poorer self-care skills arising from difficulties in undertaking the ongoing regulatory activities required for successful diabetes self-management. Lin et al. found for example, that people with co-morbid major depression and type 2 diabetes were less likely to undertake physical exercise; had unhealthier diets; and lower medication adherence [15]. Conversely, Kreyenbuhl et al. in a study comparing adherence to oral hypoglycaemic medication among veterans with and without comorbid schizophrenia, found greater adherence among people with schizophrenia which they associate with more regular outpatients appointments [16].

Social support has been identified as a significant factor in the successful adoption of lifestyle change for people with type 2 diabetes. Ashida and Heaney characterise the support provided by social networks as occurring in two ways: either through social support or social connectedness [8]. Social support in this context refers to instrumental support for illness management; emotional support; informational support and appraisal support which assists self-evaluation and awareness of health status. Social connectedness, in contrast, is subjective and is relates to a perception of isolation. Social connectedness is described by Ashida and Heaney as the experience of exchanges for pleasure [8, 10]. Social networks provide social support for self-management of chronic illness in two ways: through direct support via performing activities to manage illness or through indirect support, which encourages and facilitates self-management [10]. Direct support activities may include management of medication and diet or transportation to appointments, while indirect support activities may include such activities as providing reassurance and information or purchasing healthy food [17]. Family and friends often provide direct support for dietary management through preparing recommended food [17]. This is particularly evident for men who often view dietary management as being the responsibility of wives and partners [18]. Conversely, the same family and friends may hinder dietary changes through tempting or preparing inappropriate foods leading to a potential for social isolation through avoidance of situations where dietary restrictions need to be negotiated [19]. Family and friends can also provide support for maintenance of exercise regimes through becoming an exercise partner [17] and may seek and provide information for people newly diagnosed with type 2 diabetes [20].

Mental illness has also been associated with a loss of social ties through social distancing arising from the stigma experienced by people with mental illness [21]. Parry & Pescosolido associate contact with mental health services with diminishing network size and greater turnover of network membership with remaining network members performing a greater number of support functions [22]. Social distancing through a process of social isolation and transmitted discrimination may also apply to the families and carers of people with mental illness [23]. Lack of or limited social ties have been related to poorer self-reported mental and physical health, however this relationship is stronger if the individual perceives themselves to be isolated [10]. Likewise, people with schizophrenia who lack social networks report higher levels of stigma and depression [24].

The size and composition of social networks is in turn, related to both the level and type of professional support needed. There are five types of networks identified in the literature. These are: diverse, family-centred, locally integrated; wider integrated and restricted networks [1, 9, 23, 25, 26]. Family-centred networks are based on close family members; locally integrated networks include family and neighbours while wider integrated networks are larger and primarily friendship-centred, restricted networks have minimal social ties while diverse networks include a range of personal and community connections [1, 26]. Diverse social networks are generally associated with better mental health while restricted networks are associated with depressive symptoms [9]. Diverse social networks increase access to instrumental support through the sharing and provision of information [27]. The existence of a family network is generally linked with less reliance upon formal care services but may create other emotional demands while friendship networks are less likely to provide direct support and people reliant on these networks are more likely to receive formal care services [28].

Social networks can reduce use of professional services for mental health through provision of both direct and emotional support. People with social networks who encourage or model use of mental health services are more likely to seek support for distress [7, 29]. However, social networks can have a negative referral effect through social contacts reacting negatively to symptoms and to mental health services delaying mental health help seeking behaviour [7]. Maulik et al. explore the impact of social networks upon help seeking for mental health. They found that spousal support and regular contact with friends was associated with greater use of general medical services for mental health but decreased use of specialist mental health services. This occurs as spousal and family support promotes referral to medical services but reduces the need for specialist mental health services through providing another avenue for emotional support [7, 30]. Conversely, restricted social networks are associated with more frequent hospitalisation and subsequent loss of social support as network members are lost or replaced by people with mental illness [31]. People with regular contact with friends were found to be more likely to use other human services. This was explained by the information about the range of available services that was gained through more superficial relationships or weak ties. While these ties may not provide direct support they are a source of information that enables the individual to seek assistance [7].

This paper focuses upon the support provided by formal [paid and professional carers) and informal networks to people with mental health problems and type 2 diabetes, comparing the experiences of those people with a network containing a spouse with those without. Given that people with mental illness often experience smaller informal social networks, the impact of access to informal support upon use of formal professional services is also explored. Data are presented about the nature of the social networks of these participants and on the impact of the presence and absence of a spouse on use of other informal/unpaid and formal/paid professional support. The paper also investigates which members of a social network undertake tasks to support individuals managing physical and mental illnesses, informing understanding of the needs of people living in the community with mental illness and type 2 diabetes comorbidities.

## Methods

### Data collection

Data for this study were collected through semi-structured interviews with 29 people with a diagnosed mental illness and type 2 diabetes mellitus. The number of participants was dictated by the availability of people who met the inclusion criteria outlined below who were willing to participate in the study. The majority of participants were interviewed in their own home however three participants were recruited through a clinic seeing homeless clients and were seen within the clinic. The method for collection of social network data was based on a successful study undertaken in Manchester with people living with chronic illness in the community [32]. Participants were asked to complete an egocentric social network map which involved mapping the people and services connected to a central actor, in this case the person with mental illness and type 2 diabetes [14]. To map networks, the researchers utilised a large, portable (see Additional file 1) whiteboard upon which movable squares of paper were placed as the participants identified individuals who made up their social network. As we were interested in relationships between members of the network e.g. networks of networks, rather than relationships dyads which centre on pairs of individuals [33] participants were asked to indicate which members of the network knew each other. Additional information was collected about the participant’s perception of the value placed on each person in their network [8, 32]. In order to gain this information, the research team used print outs of money images and asked the participant to indicate importance by allocating an amount between three clear categories: highest importance ($100), middle importance ($50) and lowest importance ($20). This strategy was used as a heuristic device for participants to place relative importance on network members. Demographic data was also collected as was data about co-morbidities and services use within the last 6 months including hospitalisation. In addition, participants were asked a series of open-ended questions about self-management practices and who supports self-management. All interviews were audio recorded and a transcription made of the interviews, the completed network maps were photographed and additional notes made by a second researcher. Data was then entered into a spreadsheet.

### Recruitment

Recruitment occurred through a community nursing service. A clinician (SG) was employed on a fractional basis to screen the nursing services clinical record system to identify potential participants; contact both the participant and their regular nursing staff to provide the information sheet; and contact the participant a week later to ascertain their interest in involvement. Participants were eligible for inclusion in the study if they had a dual diagnosis of mental illness and type 2 diabetes and were in current receipt of nursing services. They were excluded if they had been diagnosed with conditions such as dementia, autism and intellectual disabilities which may compromise ability to remember or communicate the information required. Once an expression of interest was gained from the participant their contact details were forwarded to the research team who organised a time for the interview. The time was communicated to the clinician who contacted the participant on the day before the interview to ensure that they were still available and interested in being involved. This strategy was developed to enhance access but also to protect the clients of the service who were viewed as being a vulnerable population. Once the interview was completed the clinician was notified and she rang in the following week to ensure that the participant was satisfied with the interview process.

### Data analysis

Luke and Harris identify 3 approaches to analysing network data: network visualisation; descriptive analysis of network properties; and development of longitudinal and inferential models [34]. Given sample size for this study this paper draws upon the first two methods only. Analysis was undertaken through the entering of network data into UCINET software to create de-identified network maps [35]. In addition, mean network size for people with and without a spouse was calculated along with the proportion of strong, medium and weak social links identified by the importance placed on these ties by both groups. Data on who participants identified having strong links with was also collated. A second source of data came from the interview transcripts. The transcripts were analysed using the principles of grounded theory which seeks to provide a depiction of reality through allowing the theory to emerge from the data [36]. The data were initially coded using open codes which identify concepts and their properties and later subject to axial coding which makes links between the concepts [36]. Each member of the research team read three transcripts and identified themes which were then consolidated into an analytic framework developed by two members of the team (MC and JH). This framework was used as the basis for analysis using NVivo 10 [37]. This paper draws upon two aspects of the interview data: who is providing support (formal or informal networks) and the type of support provided. The data is utilised to illustrate differences between participants in relation to need for formal support on the basis of and availability of spousal and informal support.

### Ethical considerations

Ethics approval for this project was gained through Flinders University Social and Behavioural Ethics Committee. The community nursing service did not have an ethics committee but required that participant paperwork be vetted by the consumer reference group of the community nursing services before being submitted for ethics approval. Written consent was obtained from all participants prior to data collection. For the purposes of this paper, all participants have been given a pseudonym and are only referred to by this name.

## Results

Table 1 provides an overview of the participants involved in this study, listing the co-morbid conditions identified by participants. This method was adopted to protect the privacy of clinical records. While all participants had type 2 diabetes and a diagnosed mental illness this was not always reflected in conditions identified by participants. Many participants did not identify a mental health diagnosis. This may reflect the stigma associated with mental illness particularly for older people or alternately a focus upon the physical health conditions managed by the nursing service. Participants’ ages range from 32 to 94 years of age with a mean age of 64 years. Nine participants live with a spouse and 12 live alone, the remainder live in some form of supported housing (N = 4), with friends or in share houses (N = 3) and one with other family members. The number of people identified in participants’ networks ranged from 4 to 14. The mean number of contacts was 8.65 (SD ± 2.29), the majority of which were formal or paid professional contacts (5.31; SD ± 2.19). The mean number of informal social contacts was 3.34 (SD ± 1.60). Ashida and Heaney in comparable research with older people who were not restricted due to chronic illness found that the mean network size was 12 suggesting that these participants experienced more restricted networks [8]. Network size has been found to be protective of health and well-being among older adults as social relationships buffers against life stressors [38]. The vast majority of social contacts reported were with spouses or relatives rather than friends, suggesting that family provide a major source of social support and social connection. Whilst some listed neighbours in their social networks, these contacts tended to be acquaintances rather than friends and relationships were superficial. Despite this, these relationships were described as providing a source of social connection in the absence of family and friend networks and were often important.

**Table 1:** Participant summary table

Pseudonym	Age	Living Arrangements	Other chronic illnesses identified by participants	Number of people in network
Annabelle	66	Lives with spouse	Joint problems, Meniere’s syndrome, asthma, anxiety	8 (3 professional, 5 social)
Bonnie	77	Lives with spouse	Bowel issues, back problems, asthma	8 (5 professional, 3 social)
Florence	85	Residential care	Leg pain, swollen knees, back pain, leg ulcer/ft ulcer, depression.	5 (2 professional, 3 social)
Rupert	76	Lives with spouse	Heart issues (bypass), pacemaker, lung problems (asbestosis)	9 (3 professional, 6 social, 1 pet)
Maxwell	52	Homeless shelter	High blood pressure	4 (all professional)
Jack	53	Homeless shelter	Depression, high blood pressure, high cholesterol	11 (6 professional, 5 social)
Rosemary	72	Lives alone	Leg amputation, kidney problems, anxiety	14 (8 professional, 6 social)
Kate	49	Lives with ex-spouse and children	Peripheral neuropathy, cellulitis, high blood pressure, breast abscess, high cholesterol and thyroid problems	9 (3 professional, 6 social)
Alexander	52	Lives alone	Schizophrenia, Rotor Syndrome, Parkinson’s disease, arthritis, anxiety, chronic stress, bipolar, alcoholism	8 (4 professional, 4 social and 1 pet)
Robert	51	Lives alone	Epilepsy, schizoaffective psychosis, anxiety	10 (7 professional, 3 social)
William	69	Lives alone	Sleep apnoea, asbestosis, glaucoma, depression and bipolar	9 (8 professional, 1 social)
Miranda	94	Lives alone	High blood sugar, heart problems, cancer (breast)	9 (3 professional, 6 social)
Stanley	78	Lives alone	Depression, heart problems, memory problems	11 (8 professional, 3 social, 1 pet)
Sophie	64	Lives with spouse	Legally blind, arthritis, bipolar, thyroid problems	5 (4 professional, 1 social, 1 pet)
Emily	32	Lives with housemate	Stroke, arthritis, shortness of breath, depression and anxiety	9 (6 professional, 3 social 1 pet)
Penelope	44	Lives alone	Breast abscesses, obsessive compulsive disorder, sleep apnoea, depression	10 (6 professional, 4 social, 1 pet)
Jacqueline	62	Lives with spouse	Cardiovascular disease, agoraphobia, panic attacks	9 (6 professional, 3 social)
George	59	Lives alone	Depression, stroke, memory problems.	4 (1 professional, 3 social, 2 pets)
Peter	85	Lives in hostel	Heart problems (triple bypass), high blood pressure, anxiety	9 (7 professional, 2 social)
Patrick	79	Lives with spouse	Kidney problems (on dialysis) depression	9 (6 professional, 3 social)
Edward	64	Lives alone	Depression, osteoarthritis	9 (6 professional, 3 social, 1 pet)
Beatrice	79	Lives with spouse	Depression, high blood pressure, osteoporosis, arthritis	7 (4 professional, 3 social)
Richard	56	Lives with spouse	History of TIAs, high blood pressure	8 (6 professional, 2 social, 2 pets)
Libby	56	Boards with friend	Back pain, chronic pain, amputated toe, peripheral neuropathy sleep apnoea, suicidal tendencies, anxiety, depression	7 (5 professional, 2 social, 1 pet)
Ben	71	Lives alone	Dépression, leg amputation, infections, glaucoma	11 (10 professional, 1 social)
Felix	73	Lives with spouse	Retinal detachment, joint replacement, depression, hypertension, kidney problems, arthritis	9 (4 professional, 5 social, 3 pets)
Cameron	55	Lives with housemate	Kidney problems, crippling skin condition (unknown cause, not responding to treatment, needs to stay indoors), depression	7 (3 professional, 4 social)
James	78	Lives alone	Depression, anxiety, dementia, high blood pressure	11 (7 professional, 4 social)
Alexandra	53	Lives alone	Kidney disease, liver disease, high cholesterol, depression	12 (9 professional, 3 social)

Data were analysed to explore the differences between networks containing a spouse or partner and those without. People living with a spouse have been found to have larger social networks [24]. In this study, people living with a spouse experienced smaller social networks than those without a spouse (see Table 2). Participants with a spouse had slightly larger informal networks than participants without a spouse. The lack of informal supports experienced by our participants reflects limited friendship networks and reliance upon family and neighbours for support with people without a spouse identified fewer strong and a greater number of weak ties than those with a spouse (see Table 2). People with a spouse had less formal support workers than those without a spouse. Spousal networks were also denser where density refers to the numbers of ties evident divided by the number of possible ties and less centralised than the networks of people without a spouse [39]. Centrality in this context refers to the most extensively linked actors in the network with higher centralisation index scores indicating greater network centralisation [39].

**Table 2:** Comparison of networks with and without a spouse

	Network with spouse	Network without spouse
Mean number of ties	3.44	3.30
Informal	SD ± 1.59	SD ± 1.66
Formal	4.55	5.65
Total ties	SD ± 1.24	SD ± 2.46
	7.89	8.95
	SD ± 1.27	SD ± 2.58
Number of ties of each strength	43 (57 %)	82 (47 %)
Strong	21 (28 %)	58 (34 %)
Medium weak	11 (15 %)	33 (19 %)
Range of network centralisation index scores	6.6–60.8 %	12.5–90.1 %

**Fig. 1:**
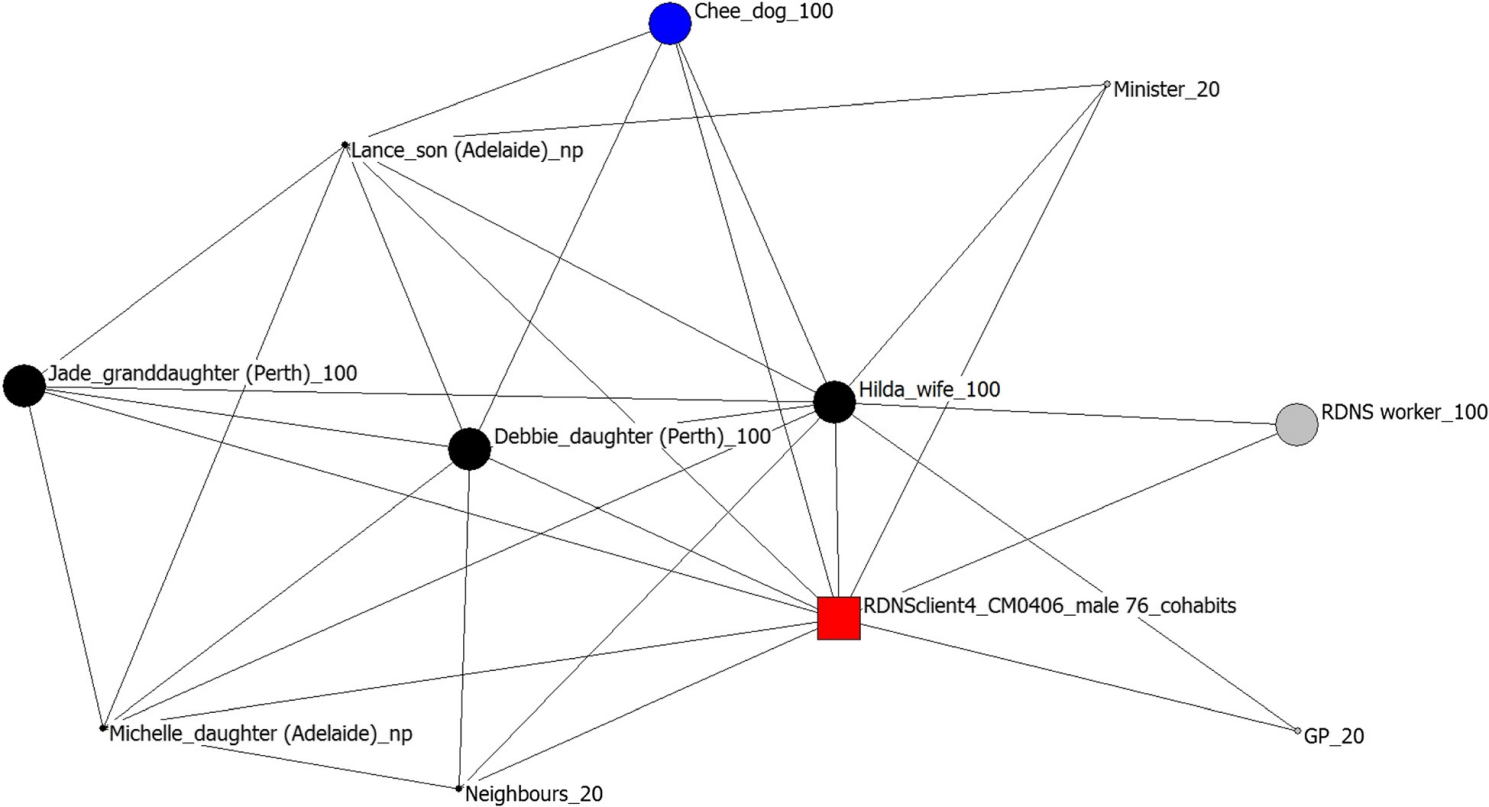
Network map of a participant with spouse and family support

**Fig. 2:**
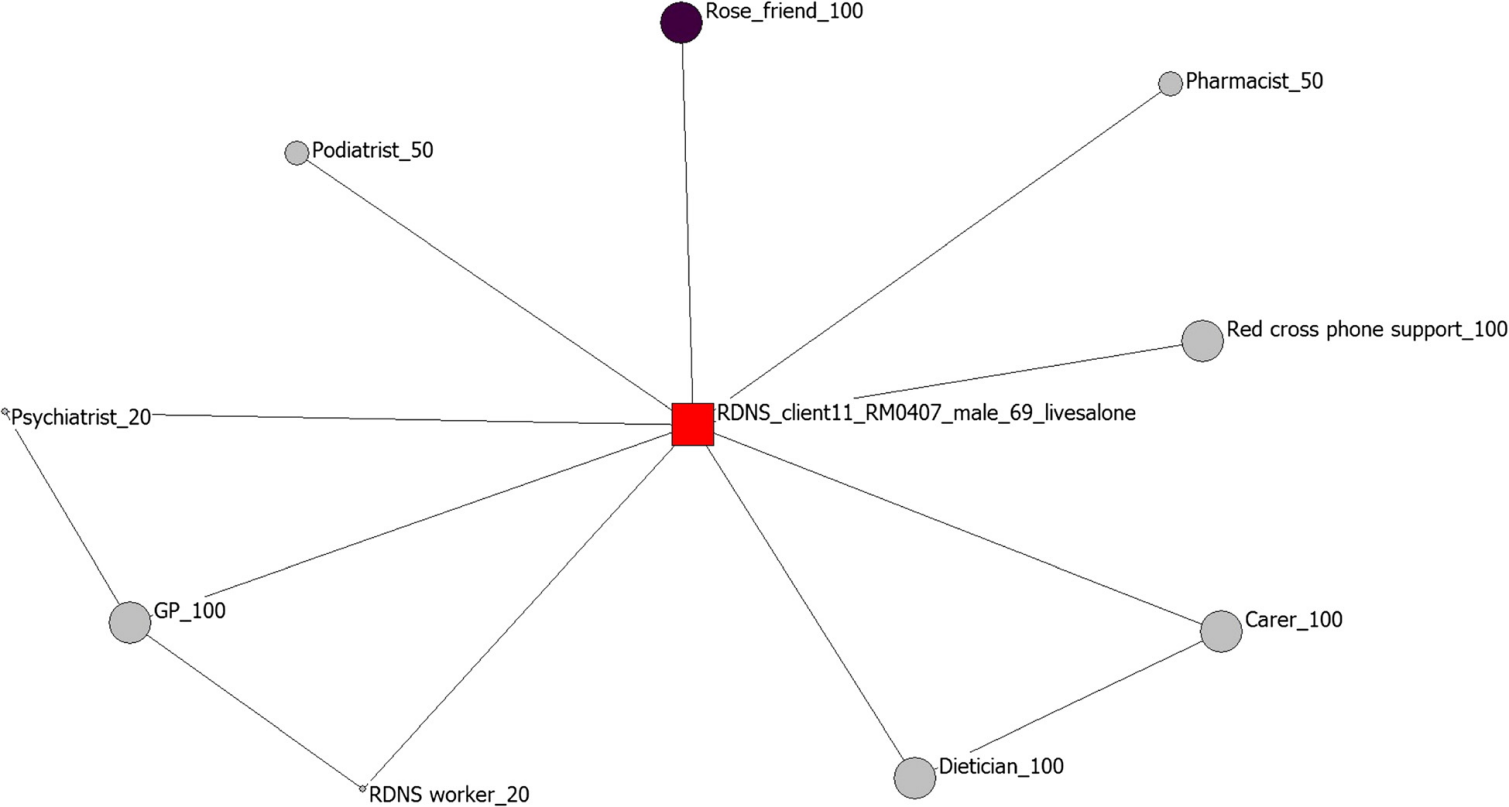
Network map of a participant without spouse or family support

Egocentric networks are by definition focussed around one person, however in spousal networks in this study, the spouse often interacts with professional caregivers and can be instrumental in maintaining social networks. The role of the spouse is also reflected in measures of betweenness. Betweenness is defined as the “number of times an actor connects other actors who would otherwise not be [connected]” [39]. These data have been analysed to determine who outside of the participant is most essential to each network. For spousal networks this is always the spouse. For networks without a spouse this role is filled by a variety of people including service providers (n = 11), other family members e.g.: children or parents (n = 7) and friends (n = 1). The role played by different network members is also reflected in the importance placed on network members (see Table 3). Participants place similar levels of importance on formal support with nursing services, medical support and personal care services important to both groups. There are differences in the importance placed on informal networks with people without a spouse placing greater importance on other family members, friends and neighbours than those with a spouse (see Table 3). The differences in network are illustrated by Figs. 1 and 2 below.

**Table 3:** Network members identified as being most important for people with and without a spouse in their network

	Spousal network	Network without spouse
Informal support		
Spouse	6 (14 %)	0 (0 %)
Other family	4 (9 %)	17 (22 %)
Friends/social groups	2 (5 %)	8 (10 %)
Pets	3 (7 %)	4 (5 %)
Neighbours	0 (0 %)	3 (4 %)
Formal support		
Community nursing service	8 (19 %)	11 (14 %)
Medical (excluding psychiatrists)	7 (16 %)	12 (15 %)
Personal care/carer	7 (16 %)	11 (14 %)
Mental health workers	1 (2 %)	5 (6 %)
Allied health	4 (9 %)	6 (8 %)
Transport	1 (2 %)	1 (1 %)

Figure 1 below shows the network of an individual with spousal/family support. As can be seen, this person has described several social contacts as well as professional support. The size of the circle reflects the importance placed upon this relationship by the respondent. This information is also conveyed by the recording of dollar value at the end of each label. Formal services are indicated in grey, family members in black, friends in purple and pets in blue. This network is well connected, demonstrating that individuals within this participant’s network are known to each other with relationships with wife, daughter, granddaughter, dog and community nurse identified as the most important relationships.

By contrast, Fig. 2 demonstrates the social and professional networks of a participant who lives alone and does not have any spouse or family support. For this participant, only one social contact has been reported and the rest of the network is comprised by largely disconnected professional services. In this case relationships with formal service providers are viewed as equally important as the relationship with the social contact.

### Informal networks

The role played by informal networks was significant for participants in this study. Informal networks were comprised of spouse or partner, family members, close friends, neighbours and other incidental people and pets.

#### Role of spouse and family members

The role played by their spouse was seen to be particularly critical for many participants in terms of everyday management of their conditions. When there was a spouse in the network this person provided support for these activities, reducing the need for formal support. Bonnie described the activities undertaken by her husband:

Yes he does [help me]. He is marvellous. He does the cooking and things. Shopping….[He] is my mainstay really. (Bonnie, 77 years)

Analysis of her social network revealed that she had only three social connections within her network and relied heavily on spousal support. For other participants, a spouse assisted with medication management and the monitoring of health. The following statement is from Sophie, a woman with bipolar disorder, whose husband monitored her diabetes:

Interviewer: So what does [husband] do for you?

He reminds me to take my insulin and if I have a hypo he gives me something to eat and drink. (Sophie, 64)

The monitoring tasks described above make a significant contribution to the work of managing type 2 diabetes. A similar routine was described by Kate who lived with her ex-husband. Despite the breakdown of the marriage, her ex-husband continued to assist her with daily medication management:

The only thing that anybody does for me that is done automatically …, is that [ex-husband] puts my tablets together. He’ll get up in the morning and put in what I need for the day and at night he’ll give me what I need for the night time. That started to happen because of the neuropathy. (Kate, 49)

In some cases, women provided this support for husbands. While these men were often very appreciative of this care, they seemed to consider this to be part of a wife’s role and so less noteworthy, therefore, it was often harder to get this care acknowledged as both Rupert and Richard conveyed:

[my wife does] Lots of things I guess. All the things that she thinks are very important and maybe I don’t. (Rupert, 76)

Interviewer: Do you have carers at all or a carer coming in to help with the house?

No, my wife is the carer of me and I’ve got domiciliary care. (Richard, 56)

When participants had children, these children undertook tasks such as management of finances, food provision, organising services and transport as well as social outings but they did not provide support for daily care management tasks. Patrick who speaks English as a second language described the role of his daughter:

I can’t, you know, write in English and she got to take everything to send to doctors and specialists and…

Interviewer: So she talks to all the people for you?

All of these people, yes (Patrick, 79)

Rosemary stated that her son problem solves “If anything goes wrong or if I need money out my bank” (Rosemary, 72). For others however, mental illness has been associated with alienation from children and other family members. This is most evident among men whose marriages had broken down resulting in alienation from their children. Edward stated for example, that family breakdown resulted in “my daughter… [being] 30 before I actually spent my first birthday with her” (Edward, 64).

#### Valued weak tie support: neighbours and incidental people

Neighbours and incidental people were found at times to substitute for the ties provided by partners and family. The nature of work undertaken was similar but less than that of family ties [40]. While most participants described some weak ties those with limited family networks relied on neighbours and other incidental people, including social or church groups, for emotional support and connection (see Table 3). In this study the incidental people found to be the main contacts within participants’ social network were varied. In some cases, these people were geographically close such as next door neighbours and therefore the capacity for regular contact was high. Stanley who lives alone described a relationship with his neighbour that reflects a desire to seek out informal contact:

I’ve got a chap across the road here who I see once a morning when he comes to work and probably in the afternoon. I make a point of getting out there waving goodbye or something, you know? (Stanley, 78)

Alexander who had multiple physical and mental health problems stated that his neighbours had taken on a monitoring role of his wellbeing to the extent that an alarm would be raised if he were not seen:

Interviewer: Would you see them [the neighbours] regularly?

Yes every day or every second day. We don’t live in each other’s pockets. If they don’t see me they start looking. (Alexander, 52)

For Libby, who had experienced a recent mental health crisis, contact with an online lecturer provided an important source of support:

The day when all the kerfuffle broke out down there she was contacting me probably every couple of hours to make sure I was okay… it was scary and having – you know, if I’d brought the cops onto the scene it wouldn’t have worked but at least I knew somebody was at the end of the line. (Libby, 56)

Although they had no face-to-face contact, the lecturer played an important role in monitoring the well-being of this woman, providing a source of social connection that may have been provided by friends or family in other circumstances. As such, the role of incidental people became more important in the absence of other social networks.

#### Role of pets

Brooks et al. in a study of 300 people managing chronic illness found that pets were identified as having a role in chronic illness self-management through provision of companionship and responding to mood change but also through reinforcing capacity and in turn, identity through competence in caring for animals [41]. For several participants in this study, pets were not only an important source of emotional support but also provided structure and routine. Penelope in describing her ageing dog said:

She is my pride and joy, mate. When I’m feeling down and sometimes depressed, when she used to get up on my bed but now she comes near me, near the chair, she always makes me feel better and if she’s happy I’m happy (…) we’ve got a very strong bond together, very strong bond. (Penelope, 44)

Here, there is not only a “strong bond” but the reference to times of depression makes it clear that for Penelope her dog is undertaking the emotional work involved in chronic illness management. For Stanley who lived alone, owning birds provided him with both structure and a caring role:

Budgies, yeah. And I’ve got three… But they’re my job, you know…It gives me some security of knowing that I’ve got a job to do, you know? (Stanley, 78).

Pet ownership may also promote the development of weak ties with others. Wood et al. associate pet ownership with increased opportunities for interaction with neighbours and improved social capital [42]. Social capital in this context is defined as social engagement which fosters social participation [43]. This is particularly evident for dog owners. William described the role dogs played in improving his social networks before he became ill.

Well I used to do volunteering a lot with dogs, with pets and that, and round nursing homes. I went to [nursing home] in Melbourne a couple of times. They’ve got a big ground there – this is well before I was sick – and I used to take the dog in there and let the dog run around with the patients (William 69).

He has decided not to have another dog due to his health status and lack of support if he was hospitalised.

### Role of formal support

In networks without a spouse the everyday tasks and activities of daily living were more likely to be performed by paid service providers, such as through care packages. Rosemary talked about the services her carer provides:

She showers me, she puts all my creams on where I need it, she’ll do meals if I need them. She always does my breakfast, makes my bed, does the washing. If it needs sweeping she’ll sweep the floor. She’s very good. (Rosemary, 72).

Services relating to illness and medication management were performed by the community nurse or pharmacist, with community nursing services frequently acting as a conduit for information to other services, as Bonnie stated:

She takes my blood pressure and checks my chest and checks my sugar levels and breathing exercises. A lot. [The nurse] does a lot. And if she’s got any query then she’ll ring [the Dr] (Bonnie, 77).

Other participants had organised their own means of getting everyday tasks done, as Ben described in his relationship with a taxi driver.

See Thursday is pay day so P [taxi driver] will come round. P will come round and he’ll drop me off at the shopping bay, run away and do a couple of jobs and then come back and when I’m ready I ring him and tell him and he picks me up (Ben, 71).

This relationship is indicative of the blurring of boundaries in relationships with paid service providers. Many participants with limited social networks befriend formal care providers who then become a source of social connectedness. Ben described a similar relationship with his cleaner who provided lunch for him on the day he was interviewed.

I tend to be very loyal. When I got out of hospital she was the first cleaning lady I had. They kept sending me around all sorts of people and I kept getting nasty with them and they kept going away and then they sent her back and she doesn’t stand any nonsense (…) Well, we got to be friends over a long period of time. (Ben, 71)

Likewise, Alexandra who has experienced homelessness in the past described her relationship with a nurse who works at the homeless shelter in the city centre:

I see her if I come in here to say hello (…) I’m not actually allowed to consult with her because I’m not classed as homeless.

Interviewer: So it’s more that you just catch up with her, it’s more of a social thing?

Yeah she just makes sure my sugar’s okay and everything’s going okay.

Interviewer: So she does it on the quiet?

Yeah because if it wasn’t for her I wouldn’t have even known I had golden staph (Alexandra, 53)

As a result of blurring of boundaries individuals carrying out a formal service can begin to undertake informal support roles and be perceived by a client as a friend. In the statements above Ben described his cleaning lady as a friend and Alexandra acknowledges that the role carried out by the nurse at the clinic is “on the quiet” and not under the remit of the nurse’s professional working role.

Piercy in exploring paid formal support workers’ opinions of client/carer boundaries found that the degree of closeness between themselves and their clients was related to the client need [44]. Where a client’s needs were met by their existing informal/social network then it was acceptable for the role played by a paid worker to be informal and like a friend. However our findings suggest that informal roles by paid workers were observed in the social networks of individuals who did not have a large informal support system. This suggests that the boundaries between formal support and friendship can be blurred where the client has a need for friendship.

## Discussion

This paper has explored the social networks that support self-management of people with type 2 diabetes and mental illness, comparing the experiences of those participants with spousal support and those without. Cummings & Kropf argue that older people living with mental illness within the community have complex needs that are best met with an array of formal and informal support systems [45]. This study demonstrates that people with mental health problems and type 2 diabetes have smaller social networks and limited informal social networks to draw upon experiencing either restricted or family only networks [1, 26]. This is not an unexpected finding as both age and physcial impairment have been associated with smaller social networks [1, 9] and people with co-morbidities have been found to have smaller and weaker social networks than people with a single disorder [46] but it is problematic when optimal care depends upon the availability of an informal support network. Mental illness has been associated with smaller social networks [21, 23, 31] with hospitalisation for mental illness creating a spiral in which small networks contribute to hospitalisation and hospitalision in turn, diminishes social networks [31]. As such, people with mental illness may not only experience impoverished social networks but may also experience social network dominated by people who also have a mental illness and who may have limited capacity to provide direct support for illness management.

The type of social network that people experience has been found to impact upon the need for and use of formal care providers. Participants in this study experience either restricted or family networks centred on spousal support. Family networks have been associated with reduced use of professional support to provide direct care activities [22]. Vassilev et al. in a study of 300 people with chronic illness found that for participants with a partner, that partner may undertake activities to manage illness; everday maintenance tasks and provide emotional support. In the absence of a partner, other family members and friends may act as a substitute [47] Our participants had fewer friends to call upon and many had experienced some degree of family breakdown due to mental illness and/or homelessness. As a consequence, people without a partner had more formal and professional support services on average and relied upon them more for everyday maintenance and illlness management tasks.

Restricted social networks have been identified as leading to depression [9, 21]. In this study, the absence of or limited frienship networks contributed to social isolation and seeking of other sources of social connection. While participants were not explicitly asked about loneliness the interview data demonstrates a degree of isolation and the importance of casual and incidental relationships. Proximity is an important determinant of these relationships. Neighbours provide both a source of regular contact but also of monitoring for many participants particularly those living alone. Rogers et al. note that weak ties may be less transient and more connective in networks of people self-manging chronic illness [40]. This trend was also evident in this study with many particpants incuding neighbours in their support networks. Likewise, many particpants demonstrated a tendency towards befriending professional care givers. The process of relationship formation is affected by continuity of care, the degree of social isolation experienced by the client and gender of the care provider with friendship more likely to develop if the client and care provider are the same gender [44]. As a consequence, the person with the dual diagnosis may risk a loss of social connection when these services are withdrawn or when service providers are replaced.

### Limitations

While the sample size for this study is small preventing generalisation to a wider population, through undertaking in-depth interviews with the network analyses we have gained insight into how participants experience mental and physical health co-morbidities and the networks that assists them to manage these comorbidities. The participants were a convenience sample recruited through a community nursing service. As such, they are in receipt of health services, with the nursing service adopting a case management role for some participants. This profile may not be typical for the population experiencing mental health and physical co-morbidities. The National Mental Health survey conducted in Australia in 2007, surveyed 855 people who consulted a health professional for mental health support within the previous 12 months. Of these people 45.6 % identified some level of unmet or partially met need, most commonly in relation to social interventions, which are defined as support with everyday tasks (64.7 %) and in skill training for work and self-care (51.3 %) [48]. This level of unmet needs suggests difficulties in attracting support for activities that are important for successful chronic illness self-management. Our sample may in fact, be better supported than many other people experiencing dual diagnoses yet they still experience a degree of deprivation.

Finally, the study focuses upon the impact of composition of social networks on self-management. The literature suggests that perception of isolation may be an important factor in maintenance of mental health and may be an intermediary factor in the relationship between small networks and diminishing mental health [7, 9]. While this relationship is explored in the interviews it is not systematically explored. This limitation may be overcome by the inclusion of survey questions about perception of isolation.

Despite limitations the study adds to knowledge through applying social networks methods to the study of the networks of people with mental illness. While the impact of mental illness on social networks is known we found surprisingly few studies applying these methods to identify the social networks these people use. Secondly, there is a dearth of information about how mental and physical co-morbidities interact and the supports people with these co-morbidities need. Finally, this study reasserts the role of informal care and highlights the paucity of informal networks experienced by participants with mental illness.

## Conclusion

This study has explored the way in which relationships identified within one’s social network can contribute to the health, wellbeing and companionship experienced by a person facing chronic comorbid diabetes and mental illness. These people in our study had more restricted and diminished social networks. Where there was a spouse then this person was a main connector in the network but within a network with a smaller number of formal service providers. A spouse helped with everyday management tasks, health monitoring and medication management. Those without a spouse and limited family relied on neighbours and other incidental people. They also relied on formal service providers who performed a wide range of tasks beyond their formal role and who, in the eyes of the client, blurred the boundary between formal helper and friend.

Given the link between social connections and mental health, the isolation experienced by those in our study without a spouse would appear to place them at greater mental health due to loss of networks. The importance of formal helpers in providing social connection and self-management support was apparent in our study, but it remains to be established if they have the capacity to provide this support over a sustained period and how more “usual” informal network supports might be promoted.

## Additional file

Additional file 1:
**Interview schedule.**
