# Supplementary material for: Analysis of social networks supporting the self-management of type 2 diabetes for people with mental illness
Source: BMC Health Serv Res. 2015 Jul 4;15:257. doi: 10.1186/s12913-015-0897-x (PMC4490681; doi:10.1186/s12913-015-0897-x)
Supplement: Additional file 1: — Interview schedule. [file 12913_2015_897_MOESM1_ESM.docx]

**Preamble: *Thanks for being part of this research study. We are interested in finding out about who helps you to manage your health conditions, in terms of individuals, groups, healthcare professionals and other people/groups. I’m going to start by asking you a few background questions, and then we will make a list of people and groups that you consider to be important in helping you to manage your health problems, and then I’ll ask you some questions about what role these people/groups play in helping you.***

**Background Questions:**

1. **Can you tell me for how long have you had Diabetes?**
2. **Do you have other health conditions?**
3. **How old are you?**
4. **Who do you live with?**

Live alone

Spouse/partner

Other family

Pets

**Making the list: show participant this list and write down people’s names as they answer on circles to place on laminated paper.**

| **Types of Support** | |
| --- | --- |
| **Relationships** | **Non medical professionals** |
| Spouse/Partner | **Legal agents (Police, lawyers)** |
| Son/Daughter | **Religious or spiritual leaders** |
| Grandchildren (female/male) | **Supervisors (Bosses, teachers)** |
| Mother/Father | **Carers** |
| Brothers/Sisters | **Volunteer (individuals)** |
| Relatives (female/male) |  |
| Friends (female/male) | **Medical professionals** |
| Pets | GP |
| Neighbours (female/male) | Nurse |
| Colleagues/Classmates (female/male) | Specialists (psychiatrists, podiatrists) |
| **Local and voluntary groups** | Pharmacist |
| **Support group** | Mental health case worker |
| **Lunch/Tea club** | Alternative medical |
| **Internet-based discussion group** | Dietician |
| **Sports groups** | Social worker |
| **Religious group** |  |
| **Other social groups/ethnic group** |  |

Social network diagram.

After the respondent completes the list of people in their network ask:

1. **What value of money would you give to each of these people in relation to how important you consider them to be in managing their condition?**

$100- the people who are considered by you to be most important in managing your condition

$50- the people considered by you to be somewhat important

$20- people who are important but are less important than people who would be given $50 or $100.

**2. What relationship is each of the people in the diagram to you? (write on circles)**

**3. What is roughly the distance from where you live and each one of the**

**people/groups/pets in the diagram?** Choose from the list below [Mark in the grid

below]

a) co-habiting

b) short walk

c) short drive or bus journey (up to 1 hour)

d) longer journey (that will take more than one hour)

**4. How often do you see (or talk on the phone, text, or via internet) each of**

**them and for how long?** [Mark in the grid below]

a) every day

b) at least once a week

c) at least once a month

d) at least once every couple of months

e) less often

**5. How much time do you spend with each one of them when you meet (or talk**

**on the phone, text, or via internet)?** [Mark in the grid below]

a) up to 30 minutes

b) between 30 minutes and 1 hour

c) between 1 and 2 hours

d) more then 2 hours

**6. My contact with this member of my networks [person/group/pet] is mostly:**

[Mark in the grid below]

a) Face-to-face

b) by telephone

c) by e-mail

d) other internet resource (e.g. chat room or facebook).

**Interview questions relating to social network diagram:**

4. Can you tell me what [name] helps you do to manage your diabetes?

5. How easy is it for you to get in touch with these people/find them in the first place?

6. How useful have you found being in touch with these people/services to be in managing your condition?

**Time you spend on managing your illness**

**1. Over the last 6 months, how much time do you spend every day on**

**activities that are related to managing your condition?**

Please ***circle*** as appropriate

Up to 30 min a day

30 min - 1 hour a day

1-2 hours a day

More than 2 hours a day

**2. Over the last 6 months, please estimate the total number of times you have**

**used each of the services below, during the last 6 months**

| Service: | Number of times used in last 6 months |
| --- | --- |
| Accident and Emergency department |  |
| Outpatient or day hospital |  |
| General Practitioner (at the surgery) |  |
| General Practitioner (at your home) |  |
| Practice nurse |  |
| District nurse |  |
| Mental health caseworker |  |
| Care worker |  |
| Dietician |  |
| Podiatrist |  |
| Diabetes Educator |  |
| Psychiatrist |  |
| Psychologist |  |

**3a.Have you had any overnight stays in hospital over the last 6 months?**

**3b. If yes to above, please list each overnight stay you have had in hospital over the last 6 months**

(Include Ward/Specialty & Number of nights)

_____________________________________________________________________________________________________________________________________________________________________________________________________________________________________________________________________________________________________________________________________________________________________________
